# Severe pneumococcal pneumonia: impact of new quinolones on prognosis

**DOI:** 10.1186/1471-2334-11-66

**Published:** 2011-03-15

**Authors:** David Olive, Hugues Georges, Patrick Devos, Nicolas Boussekey, Arnaud Chiche, Agnes Meybeck, Serge Alfandari, Olivier Leroy

**Affiliations:** 1 Service de Réanimation Médicale et Maladies Infectieuses. Hôpital Chatiliez. Tourcoing 59 - France; 2 Département de bio statistiques. CHRU Lille. 59 - France; 3 Service de Réanimation Médicale et Maladies Infectieuses. Hôpital Chatiliez. Tourcoing 59 - France

## Abstract

**Background:**

Most guidelines have been proposing, for more than 15 years, a β-lactam combined with either a quinolone or a macrolide as empirical, first-line therapy of severe community acquired pneumonia (CAP) requiring ICU admission. Our goal was to evaluate the outcome of patients with severe CAP, focusing on the impact of new rather than old fluoroquinolones combined with β-lactam in the empirical antimicrobial treatments.

**Methods:**

Retrospective study of consecutive patients admitted in a 16-bed general intensive care unit (ICU), between January 1996 and January 2009, for severe (Pneumonia Severity Index > or = 4) community-acquired pneumonia due to non penicillin-resistant *Streptococcus pneumoniae *and treated with a β-lactam combined with a fluoroquinolone.

**Results:**

We included 70 patients of whom 38 received a β-lactam combined with ofloxacin or ciprofloxacin and 32 combined with levofloxacin. Twenty six patients (37.1%) died in the ICU. Three independent factors associated with decreased survival in ICU were identified: septic shock on ICU admission (AOR = 10.6; 95% CI 2.87-39.3; p = 0.0004), age > 70 yrs. (AOR = 4.88; 95% CI 1.41-16.9; p = 0.01) and initial treatment with a β-lactam combined with ofloxacin or ciprofloxacin (AOR = 4.1; 95% CI 1.13-15.13; p = 0.03).

**Conclusion:**

Our results suggest that, when combined to a β-lactam, levofloxacin is associated with lower mortality than ofloxacin or ciprofloxacin in severe pneumococcal community-acquired pneumonia.

## Background

*Streptococcus pneumoniae *is the leading causative agent of community-acquired pneumonia (CAP). Despite new antimicrobial agents and advances in supportive measures, attributable mortality linked to pneumococcal pneumonia remains unchanged and dramatically high when patient are admitted in intensive care units (ICU) [1].

Most guidelines have been proposing, for more than 15 years, a combination of a β-lactam with either a quinolone or a macrolide as empirical, first-line therapy of severe CAP requiring ICU admission [2-8]. Although a recent study demonstrated combination antibiotic therapy to be associated with a higher survival rate than monotherapy in patients with severe CAP and shock [9], the rationale for this combination was not to increase efficacy but rather to routinely provide coverage of all common pathogens causing severe CAP and particularly, *S. pneumoniae *and *Legionella *species.

In our ICU, we followed until 2003 the 1991 French recommendations [2]. Most patients received an empirical therapy based on a β-lactam-fluoroquinolone combination. Before 2003, fluoroquinolones used were ofloxacin and ciprofloxacin. Levofloxacin replaced these quinolones since its 2003 addition to the hospital formulary. Such a replacement was comforted by the ERS, French and IDSA guidelines published between 2005 and 2007 [6-8]. We wished to determine outcomes of patients treated with a combination of β-lactam plus fluoroquinolone for severe pneumococcal pneumonia. This homogenous modification of severe CAP antibiotic management in our ICU gives us the further opportunity to assess the influence of a fluoroquinolone with enhanced activity against *S.pneumoniae*.

## Methods

### Patients

Firstly, we retrospectively collected all consecutive patients aged > 18 years who were admitted into our ICU (16-bed medical and surgical intensive care unit in a 450-bed general hospital) between January 1996 and January 2009 for severe community-acquired pneumonia (CAP) and who received a definite diagnosis of pneumococcal pneumonia. Secondly, we selected patients who received, as initial antibiotic treatment, a β-lactam plus a fluoroquinolone, used with an appropriate dosage by IV route. Thirdly, patients were divided into two groups according to the fluoroquinolone used, Group A for ofloxacin or ciprofloxacin, Group B for levofloxacin. The study protocol was submitted to the Institutional Review Board for University Hospital of Lille which gave an approval with waiver of informed consent, in agreement with French regulations concerning such retrospective studies.

CAP was defined by the following criteria observed at initial presentation or occurring within 48 h following hospitalization: acute onset of signs and symptoms of lower respiratory tract infection and a new pulmonary infiltrate found on the hospital admission chest radiograph. We excluded patients coming from nursing homes or hospitalized within 90 days prior to developing pneumonia or hospitalized > 48 h in general medical wards before ICU admission, and those with radiographic abnormalities attributed solely to any other known cause (i.e., pulmonary embolus, lung carcinoma or congestive heart failure). The decision for admission to our ICU was made, in all cases, by the attending physicians. However, only patients having a Pneumonia Severity Index (PSI) score ≥ 4 were included in this study [10].

*Streptococcus pneumoniae *was considered as the causative agent of CAP when a *S. pneumoniae *strain was isolated from > 1 blood culture or when validated sputum (< 10 squamous epithelial cells and > 25 polymorphonuclear cells per low-power field) or tracheobronchial aspirates cultures grew with > 10^5 ^cfu/mL *S. pneumoniae*. Patients having CAP due to a penicillin-resistant strain of *S. pneumoniae *(MIC > 2 mg/l) were excluded from our study.

Appropriate drug dosages were defined in the French recommendations as: amoxicillin > 50 mg/kg/d, cefotaxime > 50 mg/kg/d, ceftriaxone > 20 mg/kg/d, piperacillin > 200 mg/kg/d, ofloxacin = 200 mg/12 h, ciprofloxacin = 400 mg/12 h, levofloxacin = 500 mg/12 h [2,3,7]. These drug dosages for β-lactams, ofloxacin and ciprofloxacin were unchanged during the study period. Thus, doses used in both groups were similar.

### Data collection, evaluation and definition

Within 24 h of admission, all patients underwent clinical, radiological and biological tests. Briefly, we recorded age, gender, underlying clinical characteristics and initial vital signs. Chronic respiratory insufficiency was assessed combining the usual clinical and radiological criteria and the coexistence of ventilatory impairment assessed either before or after ICU stay. Immunosuppression was defined as recent use of immunosuppressant or systemic corticosteroids (i.e., prednisolone > 0.5 mg/kg/day for more than 1 month), human immunodeficiency virus infection, neutropenia (absolute neutrophil count < 1.000 cells/mm3), organ transplantation with ongoing immunosuppressant, cancer chemotherapy within the past 3 months, or asplenia. Shock was defined as a sustained (> 1 h) decrease in the systolic blood pressure of at least 40 mm Hg from baseline or a resultant systolic blood pressure < 90 mm Hg after adequate volume replacement and in the absence of any antihypertensive drug [11]. Severity of illness at admission to ICU was assessed using the Simplified Acute Physiology Score II (SAPS) II [12], the Sepsis-related Organ Failure Assessment (SOFA) score [13] and the logistic organ dysfunction (LOD) score [14]. We also calculated the PSI at ICU admission [10]. For all patients, information on the following therapeutic topics instituted within 48 hours following ICU admission was recorded: supportive measures such as mechanical ventilation or hemodialysis, use of vasopressor drugs, hydrocortisone, drotrecogin alfa (activated), or intensive insulin therapy. The effectiveness of initial antimicrobial therapy was assessed within 72 h after treatment as follows: A lack of clinical improvement 3 days after treatment initiation (worsening or persistent fever or hypothermia, worsening of pulmonary infiltrates or of respiratory function assessed by PaO_2_/FiO_2_) defined an ineffective treatment. On day 3, day 5 and day 7, body temperature, and SOFA score were determined. During the patient's stay in the ICU, occurrence of complications was recorded. We distinguished sepsis-related complications (secondary septic shock, acute respiratory distress syndrome or development of multiple organ failure), hospital-acquired lower respiratory tract (HA-LRT) superinfections and ICU-related complications (i.e., upper gastrointestinal bleeding, catheter-related infection, deep venous thrombosis and pulmonary embolism). Multiple organ failure (MOF), acute respiratory distress syndrome (ARDS) and HA-LRT were defined according to usual criteria [15-17]. Durations of mechanical ventilation, treatment with vasopressor drugs, and ICU length of stay were noted.

Finally, patient mortality was evaluated on D-15, and at the time of ICU discharge.

### Methods of analysis

Descriptive analyses were performed in order to check and resume data. Characteristics of patients in each group were compared. Continuous variables were compared using the Student's t test. Categorical variables were compared using Chi-square test or Fisher's exact test when Chi-square was not appropriate. Differences between groups were considered to be significant for variables yielding a p value < 0.05. A stepwise logistic regression including variables collected within the first 48 hours of ICU stay and associated with a p value < 0.15 in bivariate analysis was performed. Adjusted odd-ratios were computed using a logistic regression analysis including the independent predictors of mortality. The Kaplan-Meier product limit method and the log-rank test were used to construct and compare survival curves for patients in each group.

All statistical analyses were performed using the SAS Software, V9.1.

## Results

During the study period, 378 patients with severe CAP were admitted in our unit. Among them, 83 (22%) patients exhibited a severe pneumococcal pneumonia and, finally, we identified 70 patients treated with a β-lactam combined with a fluoroquinolone, including 53 men (75.7%) and 17 women (24.3%). The mean age was 63.8 ± 16.8 years. *S. pneumoniae *was identified in blood cultures in 25 patients (35.7%). Infection was polymicrobial in 18 patients (25.7%). Causative pathogens associated with *S. pneumoniae *were *Haemophilus influenzae *(n = 7), methicillin susceptible *Staphylococcus aureus *(n = 4), enterobacteriaceae (n = 4), *Streptococcus *spp. (n = 2) and *Moraxella catarrhalis *(n = 2). All pathogens were susceptible to at least one drug (β-lactam and/or fluoroquinolone) received by the patients. Thirty-eight patients (54.3%) were classified as Group A. β-lactams used were a third generation cephalosporin (n = 20; 52.6%), amoxicillin ± clavulanic acid (n = 16; 42.1%) and piperacillin-tazobactam (n = 2; 5.3%) combined with ofloxacin (n = 33; 86.8%) or ciprofloxacin (n = 5; 13.2%). Thirty-two patients (45.7%) were classified as Group B. β-lactams used were a third generation cephalosporin (n = 26; 81.3%), amoxicillin ± clavulanic acid (n = 5; 15.6%) and piperacillin-tazobactam (n = 1; 3.1%) combined with levofloxacin.

Main patients' characteristics on ICU admission are reported Table 1. Most characteristics were similar in the two groups. However, underlying chronic respiratory insufficiency and bacteremia were more frequent in Group B patients.

**Table 1:** Characteristics of patients with severe pneumococcal pneumonia on ICU admission*

Characteristics	Overall population n = 70	Group A n = 38	Group B n = 32	p
Age (years)	63.8 ± 16.8	63.5 ± 16.5	64.1 ± 17.4	0.87
Male	53 (75.7%)	30 (78.9%)	23 (71.9%)	0.49
Female	17 (24.3%)	8 (21.1%)	9 (28.1%)	
Malignancy	5 (7.1%)	3 (7.9%)	2 (6.3%)	0.70
Diabetes mellitus	11 (15.7%)	4 (10.5%)	7 (21.8%)	0.19
Chronic heart failure	11 (15.7%)	5 (13.1%)	6 (18.7%)	0.52
Chronic respiratory insufficiency	12 (17.1%)	3 (7.9%)	9 (28.1%)	0.02
Immunosuppression	13 (18.5%)	9 (23.6%)	4 (12.5%)	0.23
SAPS II	52.9 ± 19.5	53.3 ± 20.3	52.5 ± 18.9	0.86
LOD score	7.3 ± 4.0	7.6 ± 4.0	6.8 ± 4.0	0.40
SOFA score	8.3 ± 3.9	9.0 ± 4.1	7.5 ± 3.5	0.10
PSI 4	21 (30%)	14 (36.8%)	7 (21.9%)	0.17
PSI 5	49 (70%)	24 (63.2%)	25 (78.1%)	
Temperature (°C)	37.9 ± 1.5	38.2 ± 1.4	37.5 ± 1.5	0.04
ARF requiring MV	55 (78.5%)	30 (78.9%)	25 (78.1%)	0.93
Septic shock	34 (48.5%)	17 (44.7%)	17 (53.1%)	0.48
Bacteremia	25 (35.7%)	9 (23.6%)	16 (50%)	0.02

*Data are presented as No. (%) or mean ± SD

MV: mechanical ventilation; SAPS: simplified acute physiology score; LOD score: logistic organ dysfunction score: SOFA: Sepsis-related Organ Failure Assessment score; PSI: Pneumonia Severity Index; ARF: Acute Respiratory Failure.

Main therapeutics instituted during ICU stay, evolution of severity scores, and occurrence of complications are reported Table 2. The most significant differences between the two groups of patients were the more frequent use of drotrecogin alpha, intensive insulin therapy and hydrocortisone in Group B patients.

**Table 2:** Therapeutics data and evolution during ICU stay of patients with severe pneumococcal pneumonia*

Characteristics	Overall population n = 70	Group A n = 38	Group B n = 32	P
Cephalosporin in initial treatment	46 (65.7%)	20 (52.6%)	26 (81.3%)	0.01
Use of drotrecogin alpha	4 (5.7%)	0	4 (12.5%)	0.02
Intensive insulin therapy	30 (42.8%)	4 (10.5%)	26 (81.2%)	<0.0001
Use of hydrocortisone	24 (34.3%)	6 (15.7%)	18 (56.3%)	0.0004
Haemodialysis	10 (14.3%)	3 (7.8%)	7 (21.8%)	0.09
Body temperature on D3	37.4 ± 1.3	37.8 ± 1.0	36.9 ± 1.3	0.0008
SOFA score on D3	7.5 ± 4.8	8.0 ± 5.1	7.1 ± 4.5	0.48
Improvement on D3	43 (61.4%)	20 (52.6%)	23 (71.8%)	0.09
Body temperature on D5	37.5 ± 0.9	37.7 ± 0.9	37.3 ± 0.9	0.22
SOFA score on D5	6.4 ± 4.8	7.5 ± 4.9	5.5 ± 4.5	0.13
Body temperature on D7	37.4 ± 1.0	37.7 ± 0.9	37.0 ± 1.0	0.04
SOFA score on D7	6.6 ± 5.0	7.7 ± 5.0	5.6 ± 4.8	0.17
Sepsis-related complications	31 (44.3%)	16 (42.1%)	15 (46.8%)	0.68
HA-LRT superinfections	17 (24.3%)	7 (18.4%)	10 (31.2%)	0.21
ICU-related complications	12 (17.1%)	8 (21.0%)	4 (12.5%)	0.34
Duration of MV (days)	11.3 ± 14.3	11.2 ± 15.6	11.5 ± 12.9	0.93
Duration of vasopressor use (days)	3.5 ± 4.8	3.6 ± 5.6	3.3 ± 3.9	0.80
LOS in ICU (days)	14.6 ± 16.3	14.5 ± 19.0	14.6 ± 12.6	0.97
Mortality on D-15	14 (20%)	12 (31.6%)	2 (6.3%)	0.02
Mortality in ICU	26 (37.1%)	17 (44.8%)	9 (28.1%)	0.15

*Data are presented as No. (%) or mean ± SD

MV: mechanical ventilation; SAPS: simplified acute physiology score; LOD score: logistic organ dysfunction score: SOFA: Sepsis-related Organ Failure Assessment score; PSI: Pneumonia Severity Index; HA-LRT superinfections: hospital-acquired lower respiratory tract superinfections; LOS = length of stay.

On Day 15, 14 (20%) patients had died, 12 (31.6%) in Group A and 2 (6.3%) in Group B (p = 0.02).

**Figure 1:**
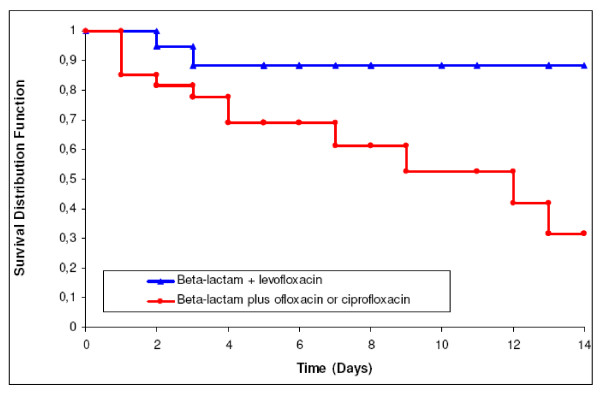
**15-day survival curves in patients treated with β-lactam combined with levofloxacin versus β-lactam combined with ofloxacin or ciprofloxacin**. Log rank test: p = 0.031

Overall, 26 patients died in the ICU, 17 (44.8%) in group A vs. 9 (28.1%) in group B (p = 0.15). So, difference in mortality rates was only significant during the first 15 days of ICU stay (Figure 1). In Group A, in-ICU mortality was 45% (9/20) when ofloxacin or ciprofloxacin were combined with a third generation cephalosporin and 44.4% (8/18) when combined with another beta-lactam, respectively (p = 0.97). In group B, it was 26.9% (7/26) when levofloxacin was combined with a third generation cephalosporin and 33.3% (2/6) when combined with another beta-lactam (p = 1).

Results of ICU-discharge survival prognosis bivariate analysis, including factors present on ICU admission, are reported Table 3. All underlying diseases (excepted chronic heart failure), mechanical ventilation, use of a third generation cephalosporin combined with a fluoroquinolone, and bacteraemia on ICU admission did not appear as significant prognostic variables in this analysis. Among the 25 bacteremic patients, mortality was higher in group A patients (66.6%) than group B patients (31.3%), but the difference was not statistically significant (6/9 vs. 5/16; p = 0.11). Among the 34 patients with septic shock on ICU admission, mortality was higher in group A patients (71%) than in Group B patients (47%), but the difference was not statistically significant (8/17 vs. 12/17; p = 0.30).

**Table 3:** Bivariate analysis of variables collected on D1 influencing the outcome, at ICU discharge, of pneumococcal CAP*

Variables	Survivors n = 44	Non survivors n = 26	p
Age > 70 yrs.	14 (31.8%)	16 (61.6%)	0.01
Gender: Male/Female	33/11	20/6	0.85
Malignancy	4 (9.0%)	1 (3.8%)	0.41
Diabetes mellitus	7 (15.5%)	4 (15.4%)	0.95
Chronic heart failure	4 (9.1%)	7 (26.9%)	0.04
Chronic respiratory insufficiency	8 (18.2%)	4 (15.4%)	0.76
Immunosuppression	10 (22.7%)	3 (11.5%)	0.24
SAPS II >50	18 (40.1%)	19 (73.1%)	0.009
LOD >8	13 (29.5%)	16 (61.6%)	0.008
SOFA >8	14 (31.8%)	18 (69.3%)	0.002
PSI 4/5	17/27	4/22	0.04
MV on D1	32 (72.7%)	23 (88.4%)	0.12
Septic shock	14 (31.8%)	20 (76.9%)	0.0003
Bacteremia	14 (31.8%)	11 (42.3%)	0.37
Patients in Group A/Group B	21/23	17/9	0.15
Cephalosporin in initial treatment	30 (68.2%)	16 (61.6%)	0.57

*Data are presented as No. (%)

MV: mechanical ventilation; SAPS: simplified acute physiology score; LOD score: logistic organ dysfunction score: SOFA: Sepsis-related Organ Failure Assessment score; PSI: Pneumonia Severity Index.

Among variables collected during the ICU stay, use of hydrocortisone, intensive insulin therapy, haemodialysis and occurrence of HA-LRT superinfections did not appear as significant prognostic variables. Conversely, improvement on D3, SOFA > 8 on D3, D5, and D7, and occurrence of sepsis-related complications were significantly associated with outcome at ICU discharge (Table 4).

**Table 4:** Bivariate analysis of variables collected during the ICU stay influencing the outcome, at ICU discharge, of pneumococcal CAP*

Variables	Survivors n = 44	Non survivors n = 26	p
Use of hydrocortisone	12 (27.3%)	12 (46.2%)	0.1
Intensive insulin therapy	18 (40.9%)	12 (46.2%)	0.66
Haemodialysis	6 (13.6%)	4 (15.4%)	0.1
Improvement on D3	36 (81.8%)	7 (26.9%)	<0.0001
SOFA >8 on D3	9 (20.5%)	14 (53.8%)	<0.0001
SOFA >8 on D5	10 (22.7%)	14 (53.8%)	0.0001
SOFA >8 on D7	10 (22.7%)	13 (50%)	0.009
Sepsis-related complications	14 (31.8%)	17 (65.4%)	0.006
HA-LRT superinfections	8 (18.2%)	9 (34.6%)	0.12

*Data are presented as No. (%)

SOFA: Sepsis-related Organ Failure Assessment score; HA-LRT superinfections: hospital-acquired lower respiratory tract superinfections.

According to the results of the bivariate analysis, the following variables were entered in the stepwise analysis: chronic heart failure, age > 70 yrs, acute respiratory failure requiring mechanical ventilation, septic shock on ICU admission, use of hydrocortisone, haemodialysis, PSI score = 5, SAPS II > 50 on D1, LOD > 8 on D1, SOFA >8 on D1 and initial treatment with a β-lactam combined with ofloxacin or ciprofloxacin (Group A).

Three independent factors associated with outcome at ICU discharge were identified: septic shock on ICU admission (AOR = 10.6; 95% CI 2.87- 39.3; p = 0.0004), age > 70 yrs. (AOR = 4.88; 95% CI 1.41-16.9; p = 0.01) and initial treatment with a β-lactam combined with ofloxacin or ciprofloxacin (AOR = 4.1; 95% CI 1.13-15.13; p = 0.03).

## Discussion

The main finding of this retrospective analysis is that levofloxacin plus a β-lactam appears to be associated with improved survival compared to ofloxacin or ciprofloxacin plus a β-lactam in severe pneumococcal CAP.

Empirical antibiotic regimen for ICU-treated severe CAP has long been recommended to cover the 3 most common severe CAP pathogens (*S. pneumoniae*, *S. aureus *and *H.influenzae*), atypical pathogens and most relevant Enterobacteriaceae species. Levofloxacin is a fluoroquinolone active against most of these pathogens, especially *S. pneumoniae *with or without decreased penicillin susceptibility [18,19]. Its clinical activity in CAP has been well documented in various clinical trials in Europe and the USA [20,21]. Some studies demonstrated the efficacy of levofloxacin used as monotherapy in severe CAP, compared to ceftriaxone plus erythromycin or cefotaxime plus ofloxacin [22,23]. Nevertheless, experts continue to propose, for ICU-treated severe CAP, an empirical antibiotic regimen based on an anti pneumococcal β-lactam combined with either a macrolide or a fluoroquinolone. Since respiratory fluoroquinolones with enhanced activity against *S. pneumoniae *(levofloxacin, moxifloxacin or gemifloxacin) became available, they replaced second generation fluoroquinolones (ofloxacin or ciprofloxacin) in the guidelines [6-8]. This fluoroquinolone generation shift has never been clearly justified and, to our knowledge, no clinical study has compared these different quinolones combined with a β-lactam in severe CAP. Our results suggest that, when severe CAP causative agent is *S. pneumoniae*, a combination levofloxacin plus β-lactam is associated with lower mortality than a combination ofloxacin or ciprofloxacin plus β-lactam.

These results could be surprising as all patients received an appropriately dosed β-lactam active against *S. pneumoniae *and as numerous strains of *S. pneumoniae *remain *in vitro *susceptible to ofloxacin or ciprofloxacin. However, there might be bacteriological and clinical data explaining our results. A synergy between β-lactams and levofloxacin against *S. pneumoniae *has been reported [24]. Conversely, synergy was rarely observed between the combination of cefotaxime and ofloxacin [25]. Recent clinical studies suggest that combination therapies could improve the prognosis of pneumococcal pneumonia: Waterer et al. retrospectively studying 225 patients with severe bacteremic pneumococcal pneumonia demonstrated that a single effective therapy was an independent predictor of mortality (AOR = 6.2) [26]. Baddour et al. performed a prospective, multicenter, international study including 844 adult patients with *S. pneumoniae *bacteremia [27]. Although the 14-day mortality was not significantly different for all patients receiving monotherapy versus combination (11.5% vs. 10.4%), a combination of *in vitro *active agents was associated with a significantly lower mortality than a single active agent (19.4% vs. 60%; p = 0.0006).

The present work has numerous limits. The most important is probably major treatment differences among the two groups. Patients were recruited during a long period (1996-2009), during which therapies such as hydrocortisone, drotrecogin alfa (activated), or intensive insulin therapy were introduced. Management of septic shock and ARDS has changed following results of large international studies [28,29]. As most changes in management of patients with multiple organ failures overlap with our antibiotic policy changes, our results might be biased. Indeed, hydrocortisone use and intensive insulin therapy were more frequent in group B than in Group A. However, these factors were not significantly associated with ICU survival in bivariate analysis and hydrocortisone use, in multivariate analysis, was not an independent prognostic factor.. Moreover, there is no evidence suggesting a survival benefit by most adjunctive therapies in patients with CAP [30] and the benefit of intensive insulin therapy in medical ICU and/or low-dose steroids is now highly questionable [31,32]. Similarly, the use of cephalosporin is more frequent in group B than in group A. However, the use of a third generation cephalosporin rather than amoxicillin has no impact on prognosis. This is not surprising as, to our knowledge, no clinical study demonstrated a third generation cephalosporin to be superior to amoxicillin for non penicillin-resistant *S. pneumoniae *CAP as far as drug dosage is adequate. Finally, some important prognostic parameters such as the time elapsed between admission and the first dose of antibiotic were not taken into account in our study. Before 2006, we did not have computerized data charts thus, exact time of admission and antibiotics admission, particularly for patients transferred from other departments/hospitals cannot be obtained.

## Conclusions

Our study suggests that levofloxacin combined with a β-lactam is associated with improved survival in comparison with ofloxacin or ciprofloxacin combined with a β-lactam in severe pneumococcal patients admitted in the ICU. This combination, proposed by current guidelines as empirical treatment of severe CAP patients admitted in ICU could improve their prognosis. Obviously, only a prospective, randomized, double-blind trial could confirm this result.

## List of abbreviations

AOR: adjusted odd ratio; ARDS: acute respiratory distress syndrome; CAP: community-acquired pneumonia; CI: confidence interval; HA-LRT superinfections: hospital-acquired lower respiratory tract superinfections; ICU: intensive care unit; LOD score: logistic organ dysfunction score; LOS: length of stay; MOF: multiple organ failure; MV: mechanical ventilation; PSI: pneumonia severity index; SAPS: simplified acute physiology score; SOFA: sepsis-related organ failure assessment; SD: standard deviation.

## Competing interests

The authors declare that they have no competing interests.

## Authors' contributions

DO collected data and helped to draft the manuscript., HG participated in the design of the study, collected data and helped to draft the manuscript, PD performed the statistical analysis., NB collected data and helped to draft the manuscript, AC collected data and helped to draft the manuscript, AM collected data and helped to draft the manuscript, SA collected data and helped to draft the manuscript, and OL contributed to the design of the study and wrote the manuscript. All authors read and approved the final manuscript.

## Pre-publication history

The pre-publication history for this paper can be accessed here:

http://www.biomedcentral.com/1471-2334/11/66/prepub

## References

[B1] Georges H Leroy O Vandenbussche C Guery B Alfandari S Tronchon L Beaucaire G Epidemiological features and prognosis of severe community-acquired pneumococcal pneumonia Intensive Care Med 1999 25 198 206 10.1007/s001340050816 10193548

[B2] Quatrième conférence de consensus en thérapeutique anti-infectieuse de la société de pathologie infectieuse de langue française Les infections des voies respiratoires Med Mal Inf 1991 21 suppl Oct 1 8

[B3] Huchon G Chidiac C Delaval P Léophonte P Mouton Y Roche N Trémolières F Management of community-acquired lower respiratory tract infection in the adult. Recommendations by the French Language Society of Pneumology with collaboration of the French Language Society of Infectious Pathology, from the recommendations of the European Respiratory Society Rev Mal Respir 1999 16 224 233 10339770

[B4] Bartlett JG Dowell SF Mandell LA File TM Jr Musher DM Fine MJ Practice guidelines for the management of community-acquired pneumonia in adults. Infectious Diseases Society of America Clin Infect Dis 2000 31 347 382 10.1086/313954 10987697 PMC7109923

[B5] Niederman MS Mandell LA Anzueto A Bass JB Broughton WA Campbell GD Dean N File T Fine MJ Gross PA Martinez F Marrie TJ Plouffe JF Ramirez J Sarosi GA Torres A Wilson R Yu VL American Thoracic Society Guidelines for the management of adults with community-acquired pneumonia. Diagnosis, assessment of severity, antimicrobial therapy, and prevention Am J Respir Crit Care Med 2001 163 1730 1754 11401897 10.1164/ajrccm.163.7.at1010

[B6] Woodhead M Blasi F Ewig S Huchon G Ieven M Ortqvist A Schaberg T Torres A van der Heijden G Verheij TJ European Respiratory Society; European Society of Clinical Microbiology and Infectious Diseases Guidelines for the management of adult lower respiratory tract infections Eur Respir J 2005 26 1138 1180 10.1183/09031936.05.00055705 16319346

[B7] XVe conférence de consensus en thérapeutique anti-infectieuse Prise en charge des infections des voies respiratoires basses de l'adulte immunocompétent Med Mal Inf 2006 36 235 244 10.1016/j.medmal.2006.04.003 16967525

[B8] Mandell LA Wunderink RG Anzueto A Bartlett JG Campbell GD Dean NC Dowell SF File TM Jr Musher DM Niederman MS Torres A Whitney CG Infectious Diseases Society of America; American Thoracic Society: Infectious Diseases Society of America/American Thoracic Society consensus guidelines on the management of community-acquired pneumonia in adults Clin Infect Dis 2007 44 Suppl 2 S27 72 10.1086/511159 17278083 PMC7107997

[B9] Rodríguez A Mendia A Sirvent JM Barcenilla F de la Torre-Prados MV Solé-Violán J Rello J CAPUCI Study Group Combination antibiotic therapy improves survival in patients with community-acquired pneumonia and shock Crit Care Med 2007 35 1493 1498 17452932 10.1097/01.CCM.0000266755.75844.05

[B10] Fine MJ Auble TE Yealy DM Hanusa BH Weissfeld LA Singer DE Coley CM Marrie TJ Kapoor WN A prediction rule to identify low-risk patients with communityacquired pneumonia N Engl J Med 1997 336 243 250 10.1056/NEJM199701233360402 8995086

[B11] Levy MM Fink MP Marshall JC Abraham E Angus D Cook D Cohen J Opal SM Vincent JL Ramsay G International Sepsis Definitions Conference 2001 SCCM/ESICM/ACCP/ATS/SIS International Sepsis Definitions Conference Intensive Care Med 2003 29 530 538 12664219 10.1007/s00134-003-1662-x

[B12] Le Gall JR Lemeshow S Saulnier F A new Simplified Acute Physiology Score (SAPS II) based on a European/North American multicenter study JAMA 1993 270 2957 2963 10.1001/jama.270.24.2957 8254858

[B13] Vincent JL Moreno R Takala J Willatts S De Mendonça A Bruining H Reinhart CK Suter PM Thijs LG The SOFA (Sepsis-related Organ Failure Assessment) score to describe organ dysfunction/failure: on behalf of the Working Group on Sepsis-Related Problems of the European Society of Intensive Care Medicine Intensive Care Med 1996 22 707 710 10.1007/BF01709751 8844239

[B14] Le Gall JR Klar J Lemeshow S Saulnier F Alberti C Artigas A Teres D The Logistic Organ Dysfunction system: a new way to assess organ dysfunction in the intensive care unit; ICU Scoring Group JAMA 1996 276 802 810 10.1001/jama.276.10.802 8769590

[B15] Tran DD Groeneveld AB van der Meulen J Nauta JJ Strack van Schijndel RJ Thijs LG Age, chronic disease, sepsis, organ system failure, and mortality in a medical intensive care unit Crit Care Med 1990 18 474 479 10.1097/00003246-199005000-00002 2328591

[B16] Bernard GR Artigas A Brigham KL Carlet J Falke K Hudson L Lamy M Legall JR Morris A Spragg R The American-European consensus conference on ARDS Am J Respir Crit Care Med 1994 149 818 824 7509706 10.1164/ajrccm.149.3.7509706

[B17] Garner JS Jarvis WR Emori TG Horan TC Hughes JM CDC definitions for nosocomial infections Am J Infect Control 1988 16 128 140 10.1016/0196-6553(88)90053-3 2841893

[B18] Davis R Bryson HM Levofloxacin. A review of its antibacterial activity, pharmacokinetics and therapeutic efficacy Drugs 1994 47 677 700 10.2165/00003495-199447040-00008 7516863

[B19] Thomson KS Chartrand SA Sanders CC Block SL In-vitro activity of levofloxacin against *Streptococcus pneumoniae *with various levels of penicillin resistance J Antimicrob Chemother 1999 43 Suppl C 15 19 10.1093/jac/43.suppl_3.15 10404332

[B20] File TM Jr Segreti J Dunbar L Player R Kohler R Williams RR Kojak C Rubin A A multicenter, randomized study comparing the efficacy and safety of intravenous and/or oral levofloxacin versus ceftriaxone and/or cefuroxime axetil in treatment of adults with community-acquired pneumonia Antimicrob Agents Chemother 1997 41 1965 1972 9303395 10.1128/aac.41.9.1965 PMC164046

[B21] Frank E Liu J Kinasewitz G Moran GJ Oross MP Olson WH Reichl V Freitag S Bahal N Wiesinger BA Tennenberg A Kahn JB A multicenter, open-label, randomized comparison of levofloxacin vs azithromycin plus ceftriaxone in hospitalized adults with moderate to severe community-acquired pneumonia Clin Therap 2002 8 1292 1308 10.1016/S0149-2918(02)80034-0 12240780

[B22] Fogarty C Siami G Kohler R File TM Tennenberg AM Olson WH Wiesinger BA Scott Marshall JA Oross M Kahn JB Multicenter, open-label, randomized study to compare the safety and efficacy of levofloxacin versus ceftriaxone sodium and erythromycin followed by clarithromycin and amoxicillin-clavulanate in the treatment of serious community-acquired pneumonia in adults Clin Infect Dis 2004 38 S16 23 10.1086/378406

[B23] Leroy O Saux P Bédos JP Caulin E for the levofloxacin study group Comparison of levofloxacin and cefotaxime combined with ofloxacin for ICU patients with community-acquired pneumonia who do not require vasopressors Chest 2005 128 172 183 10.1378/chest.128.1.172 16002932

[B24] Kühn F Cottagnoud M Acosta F Flatz L Entenza J Cottagnoud P Cefotaxime acts synergistically with levofloxacin in experimental meningitis due to penicillin resistant pneumococci and prevents selection of levofloxacin-resistant mutants in vitro Antimicrob Agents Chemother 2003 47 2487 2491 12878509 10.1128/AAC.47.8.2487-2491.2003 PMC166100

[B25] Gimeno C Borja J Navarro D Valdés L García-Barbal J García-de-Lomas J In vitro interaction between ofloxacin and cefotaxime against gram-positive and gram negative bacteria involved in serious infections Chemotherapy 1998 44 94 98 10.1159/000007098 9551238

[B26] Waterer GW Somes GW Wunderink RG Monotherapy may be suboptimal for severe bacteremic pneumococcal pneumonia Arch Intern Med 2001 161 1837 1842 10.1001/archinte.161.15.1837 11493124

[B27] Baddour LM Yu VL Klugman KP Feldman C Ortqvist A Rello J Morris AJ Luna CM Snydman DR Ko WC Chedid MB Hui DS Andremont A Chiou CC International Pneumococcal Study Group: Combination antibiotic therapy lowers mortality among severely ill patients with pneumococcal bacteremia Am J Respir Crit Care Med 2004 170 440 444 10.1164/rccm.200311-1578OC 15184200

[B28] The Acute Respiratory Distress Syndrome Network Ventilation with lower tidal volumes as compared with traditional tidal volumes for Acute Lung Injury and the Acute Respiratory Distress Syndrome N Engl J Med 2000 342 1301 1308 10.1056/NEJM200005043421801 10793162

[B29] Dellinger RP Levy MM Carlet JM Bion J Parker MM Jaeschke R Reinhart K Angus DC Brun-Buisson C Beale R Calandra T Dhainaut JF Gerlach H Harvey M Marini JJ Marshall J Ranieri M Ramsay G Sevransky J Thompson BT Townsend S Vender JS Zimmerman JL Vincent JL Surviving Sepsis Campaign: international guidelines for management of severe sepsis and septic shock Intensive Care Med 2008 34 17 60 10.1007/s00134-007-0934-2 18058085 PMC2249616

[B30] Siempos II Vardakas KZ Kopterides P Falagas ME Adjunctive therapies for community-acquired pneumonia: a systematic review J Antimicrob Chemother 2008 62 661 668 10.1093/jac/dkn283 18641037

[B31] NICE-SUGAR Study Investigators Finfer S Chittock DR Su SY Blair D Foster D Dhingra V Bellomo R Cook D Dodek P Henderson WR Hébert PC Heritier S Heyland DK McArthur C McDonald E Mitchell I Myburgh JA Norton R Potter J Robinson BG Ronco JJ Intensive versus conventional glucose control in critically ill patients N Engl J Med 2009 360 1283 1287 10.1056/NEJMoa0810625 19318384

[B32] Ferrer R Artigas A Suarez D Palencia E Levy MM Arenzana A Pérez XL Sirvent JM Edusepsis Study Group Effectiveness of treatments for severe sepsis Am J Respir Crit Care Med 2009 180 861 866 10.1164/rccm.200812-1912OC 19696442

